# The continual presence of C3d but not IgG glomerular capillary deposition in stage I idiopathic membranous nephropathy in patients receiving corticosteroid treatment

**DOI:** 10.1186/1746-1596-7-109

**Published:** 2012-08-21

**Authors:** Rui Zhang, Zhi-yong Zheng, Jian-song Lin, Li-juan Qu, Feng Zheng

**Affiliations:** 1 Department of Pathology, Dongfang Hospital, Fujian Medical University, Fuzhou 350025, China; 2 Department of Pathology, The second Hospital of Xiamen, Xiamen, 361021, China; 3 Department of Nephrology, Dongfang Hospital, Fujian Medical University, Fuzhou, 350025, China

**Keywords:** C3d, Membranous Nephropathy, Immune deposit

## Abstract

**Background:**

Pathologic diagnosis of stage I idiopathic membranous nephropathy (MN-I) requires electron microscopy or immunohistochemistry that shows a glomerular capillary staining pattern of IgG and C3. However, it is not uncommon that renal biopsy did not obtain sufficient material for electron microscopy and that IgG and C3 staining in glomeruli largely lost at biopsy due to corticosteroid treatment. Since C3d is one of the final degradation products of C3 that is more stable in vivo, we determine if C3d staining could be used as a novel immunohistochemical marker for MN-I.

**Methods and results:**

74 MN-I patients with electron microscopy proven MN-I were examined by immunoperoxidase staining of C3d. Intensive C3d staining was present in glomerular capillary like the staining pattern of IgG and C3 in MN-I. Importantly, in 40 MN-I patients who underwent corticosteroid treatment at biopsy the intensity and glomerular capillary pattern of C3d staining remained largely intact while the staining for IgG had substantially reduced and the pattern of glomerular capillary staining became unrecognizable.

**Conclusions:**

C3d glomerular capillary staining may be a novel marker for pathologic diagnosis of MN-I that is continuously present at biopsy in patient who has received corticosteroid treatment.

**Virtual slides:**

The virtual slide(s) for this article can be found here: http://www.diagnosticpathology.diagnomx.eu/vs/2120780075734479

## Background

Pathologic diagnosis of stage I idiopathic membranous nephropathy (MN-I) requires electron microscopy since the disease exhibits minimal glomerular change under optical microscopy. However, in clinical practice, it is not uncommon that biopsy materials are not sufficient to process for electron microscopy. Immunofluorescence or immunoperoxidase staining for immunoglobulin G (IgG) and complement 3 (C3), which show a typical pattern of capillary staining for membranous nephropathy (MN), has been recommended in the absence of electron microscopy to help for differential and final diagnosis. The problem is that corticosteroid treatment usually have been initiated in most of patients before biopsy and the treatment often quickly leads to substantial decrease or disappear of immune depositions of IgG and C3. Thus, it is important to find another immune deposit marker that shows the same capillary pattern as IgG and C3 in MN, but remains detectable at biopsy even patients have started corticosteroid therapy. Activation of complement pathways plays a critical role in podocyte injury in MN. C3 is the most abundant complement protein that is essential at both classical and alternative complement pathways. C3 becomes activated after it is cut to C3a and C3b two parts by C3 convertase(s). Inactivation of C3b by its degradation enzymes results in the formation of C3c and the final product C3d. Immunostaining of C3d has been widely applied to renal and liver transplant biopsies to help for acute rejection diagnosis [1-4]. An increase in C3d mesangial staining has also been reported in IgA nephropathy [5-7]. Additionally, there was a report of positive C3d staining in glomeruli of patients with MN [8]. Since our preliminary examination showed the presence of capillary C3d immunostaining like C3 and IgG in glomeruli of MN-I, we further examined if C3d could be used as an immune deposit marker for MN-I even after patients have been treated for a period with corticosteroid.

## Methods

### Case selection

Our department received 5110 renal biopsy specimes between June 2009 and July 2010 from 20 hospitals around China. The study was approved by the hospital’s Ethics and Research Committee. MN was diagnosed in 303 cases (5.9%) and 74 cases (24.4%) were MN-I. Among 74 MN-I, 40 patients have started corticosteroid therapy before biopsy (Group A) while 34 patients had not (Group B). 20 cases of mild mesangioproliferative glomerulonephritis (m-MsPGN) (Group C) and 10 cases of minimal change disease (MCD) were selected as controls for studying immunostaining pattern of C3d. Normal kidney sections were also examined.

### Pathologic criteria

The renal biopsies were examined by optical microscopy, immunohistochemistry and electron microscopy. Sections for optical microscopy were stained with Haematoxylin and Eosin, Periodic acid–Schiff (PAS), Periodic acid-silver Methenamine and Masson’s Trichrom (PAM-Masson) stains. A minimal of eight glomeruli was required for pathological examination. The diagnosis of MN-I included minimal change in glomerular basement membranes under optical microscopy and IgG immunostaining showing a typical pattern of capillary staining and focal foot process effacement and focal subepithelial immune deposit under electron microscopy.

### Immunohistochemistry

Immunostaining for C3d was performed using the EliVision^TM^ system (Maixin. Biocompany, Fuzhou China). Brifely, deparaffinized sections were put in stainless steel pressure cooker with citrate buffer (pH 6.0) heating for antigen retrieval, following with 0.1% trypsin (Becton Dickinson and Company, USA) digestion for 90 s. Endogenous peroxidase activity was blocked with 3% hydrogen peroxide for 10 min. Tissues then were incubated with first antibody, rabbit polyclonal against C3d (ab15981, 1:1000, abcam, USA) for 2 h. After reacting with a polymer enhancer at 37°C for 20 min, tissues were incubated with a secondary anbtibody, an anti-rabbit immunoglobulin conjugated with peroxidase-labeled dextran polymer (Maixin Biocompany, Fuzhou, China) 37°C for 30 min. Positive staining was revealed by immersing the sections in an aminoethyl carbazole substrate solution (ZSGB Biocompany, Fuzhou, China), followed by nuclear staining with hematoxylin [9].

In order to confirm the effect of using high-pressure heating and trypsin for antigen retrieval in C3d immunohistochemical staining, we chose serial sections of paraffin-embedded tissue of stage II idiopathic membranous nephropathy (MN-II) with no retrieval, either high-pressure heating or trypsin retrieval and high-pressure heating plus trypsin retrieval, respectively. Negative control was set in each group and primary antibody was replaced by phosphate-buffered saline (PBS).

Since C3c is another degradation product of C3b, we also performed immunostaining for C3c to compare with C3d for staining pattern and their presence after corticosteroid treatment. Immunostaining for IgG, IgM, IgA, and C1q were also performed. A semiquantitative measurement of the staining area was performed. A score of 0 to 3 was defined as: 0, no staining (negative); 1, < 25% of glomerulus stained (+, weak); 2, 25% to 50% glomerulus stained (++, moderate); 3, >50% glomerulus stained (+++, strong) [4,10,11].

### Statistical analysis

Values are expressed as mean ± SD. Results were analyzed by nonparametric test, chi-square test and Fisher’s exact probability test using SPSS 16.0 software. A *P* value of less than 0.05 was considered statistical significance.

## Results

### Characteristics of patients and renal pathology

Demographic profile such as age and sex and clinical presentations such as proteinuria were similar between MN-I patients treated without or with corticosteroid before biopsy (Table 1). Similar amount of proteinuria between treated and untreated patients suggests that corticosteroid treatment had not been effective at the time. Clinical features of m-MsPGN patients were also listed on Table 1. Histological findings for MN-I patients included slight dilatation of the glomerular capillary lumens with basement membranes showing slightly stiffness but normal thickness. Podocytes appeared swollen. Under transmission electron microscope, there were sparse, minute, subepithelial deposits. Focal foot process effacement could be seen (data not shown).

**Table 1:** Clinic characteristics of the three groups of patients

	**Group A (*n*** **= 40)**	**Group B (*n*** **= 34)**	**Group C (*n*** **= 20)**
Sex (male/female)	20/20	17/17	12/8
Age (years)	45.50 ± 14.82	46.79 ± 13.62	24.95 ± 14.10
Time to biopsy (days)	113.90 ± 105.46	55.03 ± 63.67	59.85 ± 78.60
Steroid treatment (%)	100	0	65
MAP (mmHg)	94.45 ± 10.00	92.78 ± 10.81	88.42 ± 10.23
Hematuria (%)	0	0	5
Proteinuria (g/24 h)	5.80 ± 3.15	4.21 ± 2.37	3.72 ± 3.47
SCr(umol/L)	74.89 ± 26.42	59.34 ± 13.03	77.01 ± 37.94
BUN(mmol/L)	5.27 ± 1.59	3.89 ± 1.06	5.37 ± 1.88

Group A: patients treated with corticosteroid before biopsy.

Group B: patients treated without corticosteroid before biopsy.

Group C: patients with mild mesangioproliferative glomerulonephritis.

#### C3d

**Figure 1:**
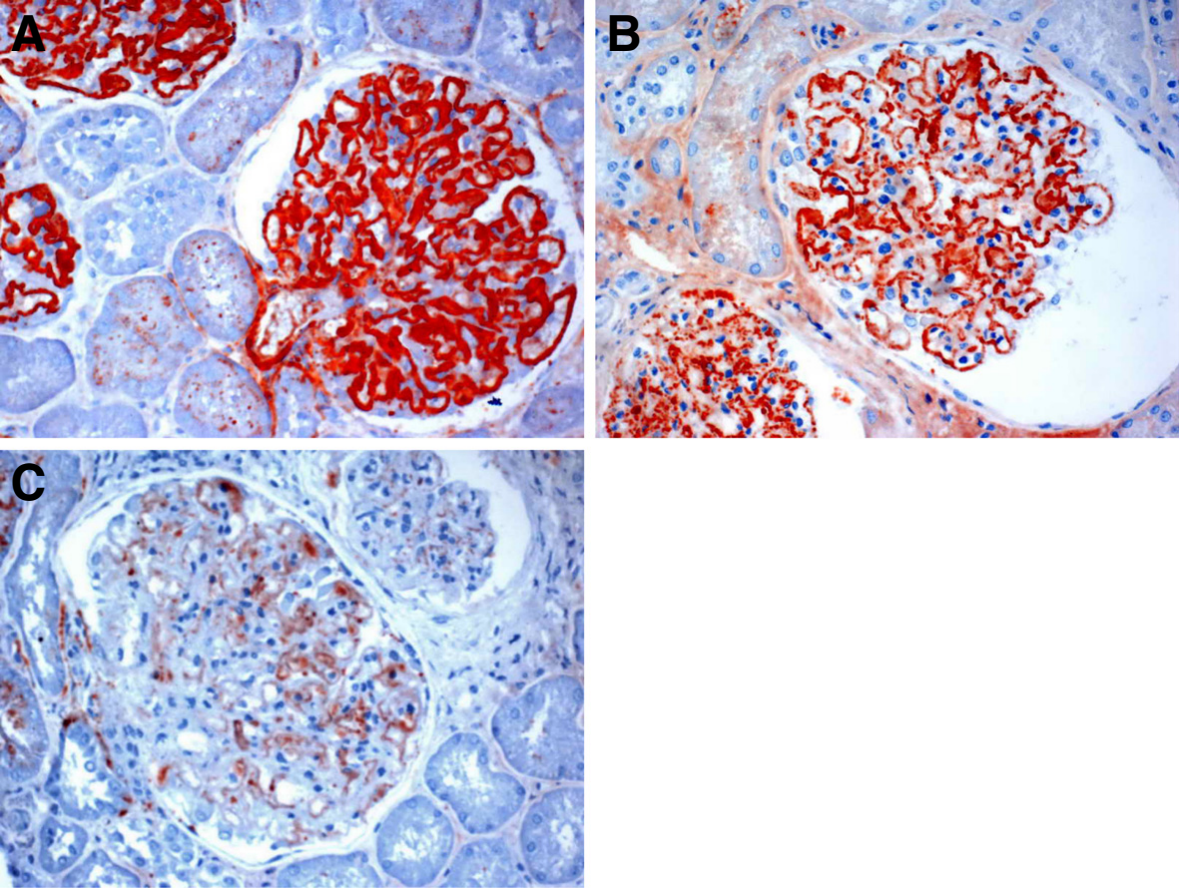
**Glomerular C3d, IgG and C3c staining in MN-I. A**, A strong C3d staining was universally present in glomerular capillary. **B**, The IgG staining in glomeruli showed a predominantly capillary pattern. **C**, Staining for C3c showing similar capillary pattern but much lesser intensity.

Deposition of C3d in glomeruli was examined by immunoperoixdase staining. C3d staining was essentially negative in normal kidney glomeruli (data not shown). A strong C3d staining was universally present in glomeruli of MN-I patients who have not received corticosteroid therapy at biopsy (Figure1A). The staining in MN-I glomeruli showed a predominantly capillary pattern like the pattern of IgG staining (Figure1B). Immunostaining for C3c, another degradation product of C3b, showed similar capillary pattern like C3d in MN-I but was with much lesser intensity (Figure1C).

**Figure 2:**
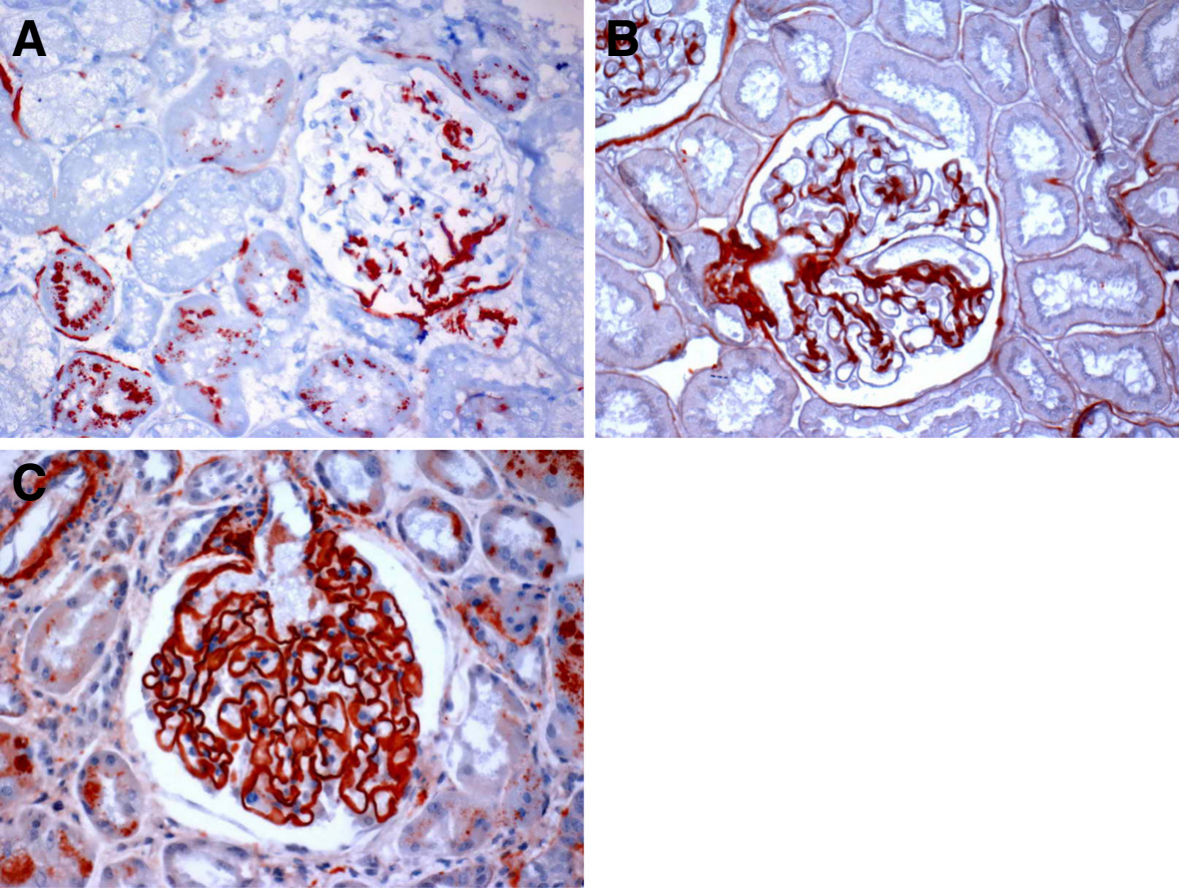
**Different pattern of glomerular C3d staining in minimal change disease, m-MsPGN and MN-I. A**, C3d staining was seen in part of mesangium in minimal change disease. **B**, The mesangial staining was more obvious in m-MsPGN. **C**, Staning for C3d showed predominantly capillary pattern in MN-I.

We further evaluated the presence and pattern of C3d staining in glomeruli of MCD and m-MsPGN. C3d staining was seen in part of mesangium in MCD (Figure2A). The mesangial staining was more obvious in m-MsPGN and the staining occasionally was extended to glomerular capillary (Figure2B). Nevertheless, the staining pattern of C3d in both MCD and m-MsPGN was mostly mesangium dominant and was clearly different from capillary pattern found in MN-I (Figure2C).

**Figure 3:**
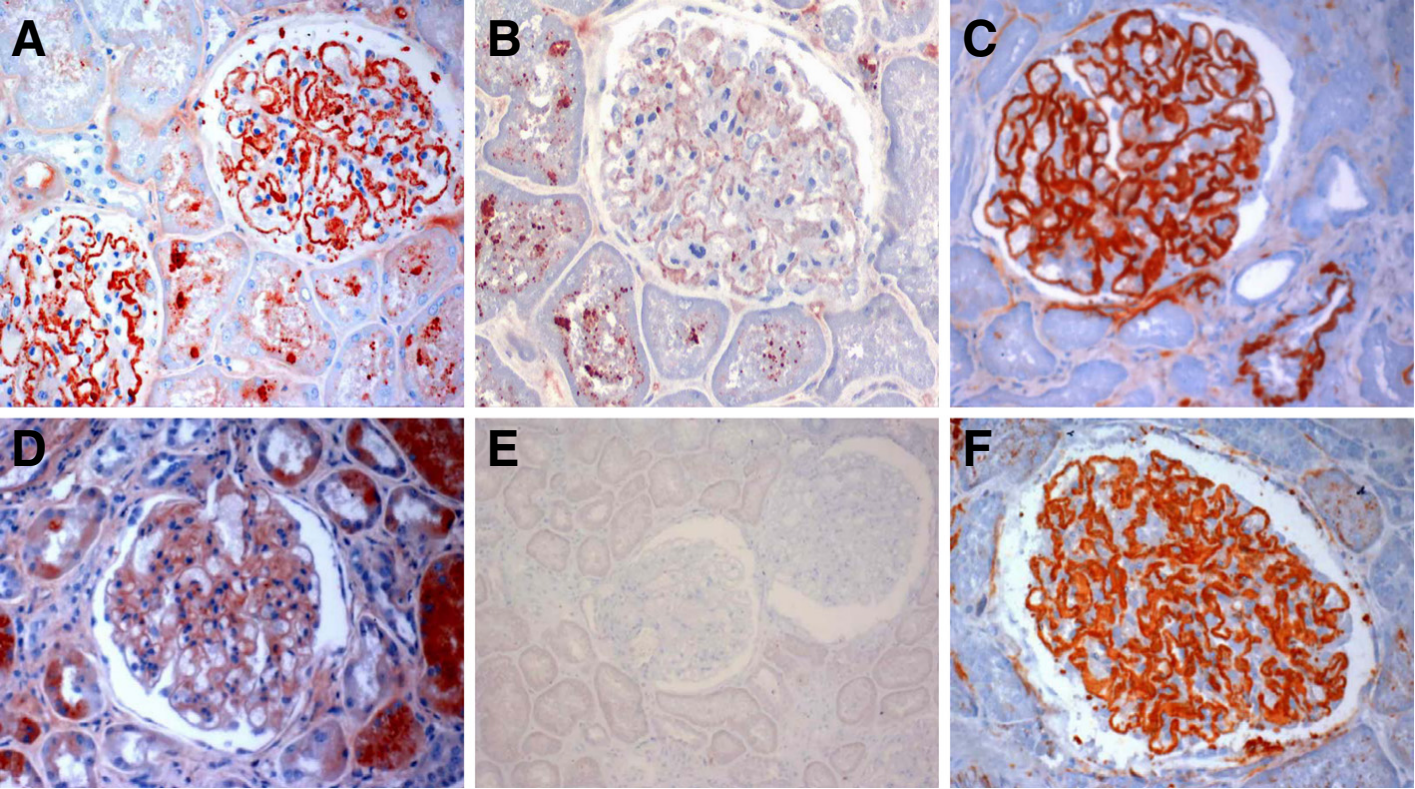
**Glomerular IgG, C3d, and C3c staining in MN-I after corticoid treatment. A**,**D**, IgG in biopsy samples from patients treated with and without corticosteroid, respectively. **B,E**, C3c staining pattern treated with and without corticosteroid, respectively. **C,F**, C3d in MN-I treated with and without corticosteroid, respectively.

It is known that glomerular staining of IgG and C3 in MN-I would largely reduce and the pattern of capillary staining became either unrecognizable or largely lost after corticosteroid therapy. To determine if this would also be the case for C3d, we compared the intensity and staining pattern of C3d in biopsy samples from patients treated with and without corticosteroid, and also compared the incidence of C3d, C3c and IgG staining. As expected, the staining for glomerular IgG in MN-I was substantially reduced and capillary pattern of staining had largely lost after corticosteroid treatment (Figure3D). C3c staining was disappeared (Figure3E). On the contrary, C3d staining remained as strong and glomerular capillary staining pattern was unchanged in MN-I patients treated with corticosteroid (Figure3F). Figure3A, Figure3B and Figure3C showed IgG, C3c and C3d staining in biopsy samples from patients treated without corticosteroid, respectively. As shown in Table 2, group A showed a significantly higher incidence of C3d and the intensity of C3d was also significantly stronger than that of IgG and C3c (*P* <0.01). Moreover, the incidence of IgG and C3c were significantly higher in group B than group A, the intensity of IgG and C3c were significantly stronger in group B, compared to group A (*P* <0.01). However, no differences for intensity and incidence of C3d were found between group A and group B.

**Table 2:** The results of immunohistochemistry examination in group A and group B

	**Positive part**	**IgG**	**IgM**	**IgA**	**C3c**	**C3d**	**C1q**
Group A (*n* = 40)	capillary lumens	0.95 ± 1.11 (52.5%)	0	0	0.03 ± 0.16 (3.33%)	3.00 ± 0.00 (100%)^a^	0
Group B (*n* = 34)	capillary lumens	2.94 ± 0.24 (100%)^b^	0	0	0.29 ± 0.58(23.5%)^b^	3.00 ± 0.00 (100%)	0

Group A: patients treated with corticosteroid before biopsy.

Group B: patients treated without corticosteroid before biopsy.

^a^*P* <0.01 *versus* IgG and C3c in the same group; ^b^*P* <0.01 *versus* group A.

### Antigen retrieval effect

**Figure 4:**
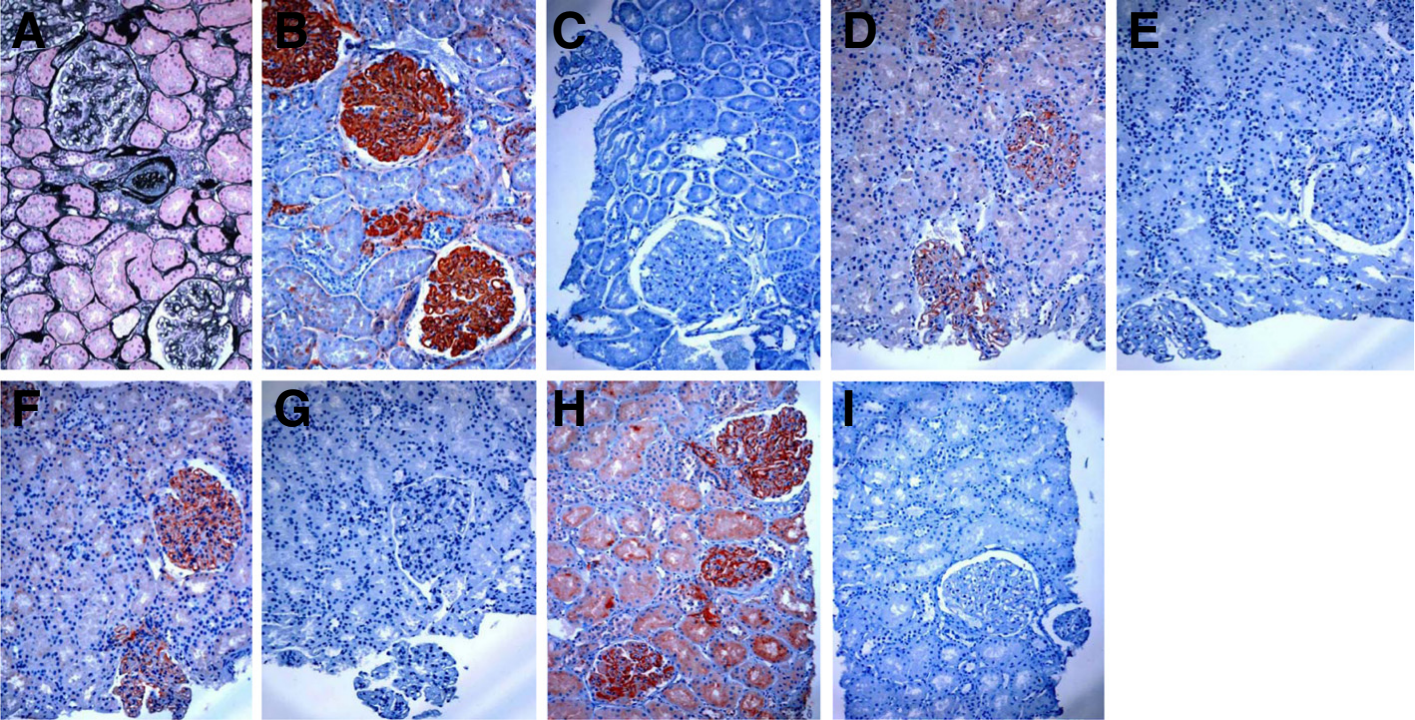
**C3d immunohistochemical staining in serial sections of paraffin-embedded tissue of MN-II. A**. PAM-Masson stain. **B**. The same region with A, with high-pressure heating plus trypsin retrieval, C3d staining was strongly positive in capillary loop, no backgroud. C3d was also positive in the global sclerotic glomerulus. **C**. With high-pressure heating plus trypsin retrieval, primary antibody was replaced by PBS as negative control. **D**. With no antigen retrieval, C3d was faintly positive. **E**. The negative control with no antigen retrieval. **F**. With trypsin retrieval, C3d was weakly positive. **G**. The negative control with trypsin retrieval. **H**.With high-pressure heating retrieval, C3d was moderately positive. **I**. The negative control with high-pressure heating retrieval.

Cases of MN-II with high-pressure heating plus trypsin retrieval, C3d staining was strongly positive in capillary loop, no backgroud (Figure4B**)**. With no antigen retrieval, C3d staining was faintly positive (Figure4D). With trypsin retrieval, C3d staining was weakly positive (Figure4F). With high-pressure heating retrieval, C3d was moderately positive (Figure4H), and Controls were negative (Figure4C, 4E, 4G and 4I). Figure4A showed the PAM-Masson stain study.

## Discussion

Due to its minor change under optical microscopy, the diagnosis of MN-I depends largely on electron microscopy. However, some biopsy materials may not be sufficient for electron microscope examination. For instance, we received 5110 renal biopsy specimens from hospitals all over the China between June 2009 and July 2010, of which 1328 cases (26.0%) did not have sufficient tissue for electron microscopy. Immune complex formation in subepithelial and resultant complement activation has been implicated in the pathogenesis of MN. A typical glomerular capillary pattern of IgG and C3 depositions is present in patients with MN. Since IgG and C3 staining are largely negative in MCD and show predominantly a pattern of mesangial staining in m-MsPGN, two diseases with minimal changes under optical microscopy like MN-I, IgG and C3 immunostaining are very helpful for differential diagnosis amongst MN-I, MCD and m-MsPGN when electron microscopy is missing. However, the staining intensity and pattern of IgG and C3 are significantly affected by corticosteroid treatment. In this study, we found an exclusively positive and strong glomerular capillary IgG staining in biopsy samples obtained from MN-I patients untreated with corticosteroid. In biopsy samples obtained from MN-I patients have been treated with corticosteroid, however, only 52.5% of tissues showed a weak IgG glomerular staining and the capillary staining pattern became largely unrecognizable in most of patients.

C3b is one of the cleavage products of C3 that plays a critical role in activating both classical and alternative pathways of complement. C3b is degraded stepwise to inactive C3b and then to C3c and C3dg. C3d is a final cleavage product of C3dg and is a stable marker of complement activation that binds covalently to cell surfacess and basement membrance, so it can persist for a long time in the tissue. C3d deposition has been found in patients with MN [7]. We examined glomerular staining of both C3c and C3d in MN-I patients. Although both C3c and C3d are final degradation products of C3b, the intensity of C3c glomerular staining was substantially weaker than C3d. This may be due to the problem of anti-C3c antibody or a shorter half-life of C3c [12,13]. Nevertheless, C3c staining is also significantly affected by corticosteroid treatment. Both intensity and incidence of C3c glomerular staining was substantially reduced in MN-I patients treated with corticosteroid at biopsy. In contrast, C3d glomerular staining was strong and showed a typical capillary pattern in MN-I and remained largely unchanged by corticosteroid therapy. Additionally, we found that the completely sclerosing glomeruli showed C3d-positive (Figure4B), which indicating that C3d could persist for a long time in the tissue and didn’t disappear. Since glomerular staining of C3d is either negative or shows a mesangial dominantly pattern in patients with MCD and m-MsPGN, C3d immunostaining could be applied as a good immune deposit marker for pathologic diagnosis of MN-I, especially in condition that patient has been treated with corticosteroid. Since corticosteroid treatment seemed largely ineffective judged by the present of intensive proteinuria in MN-I patients included in this study, it is unclear if C3d staining would remain in patients after successful corticosteroid treatment. Additionally, the molecular basis of different intensity and pattern of C3c and C3d glomerular staining in MN-I patients is unknown.

The C3d staining results of comparison showed that the use of high-pressure heating and trypsin for antigen retrieval can get the best staining results. With no antigen retrieval C3d staining was faintly positive. With trypsin retrieval C3d staining was weakly positive. With high-pressure heating retrieval C3d staining was moderately positive. Controls showed negative. These results suggested that antigen retrieval twice can improve the renal biopsy paraffin-embeded tissues by immuoperoxidase method and does not cause false positive and background staining.

## Conclusions

In summary, our study suggests that C3d glomerular capillary staining may be a novel marker for pathologic diagnosis of MN-I that is continuously present at biopsy in patient who has received corticosteroid treatment. This finding is particularly significant when there are no sufficient biopsy materials to process for electron microscopy which is considered to be the gold standard for the diagnosis of MN.

## Abbreviations

MAP: Media arterial pressure; SCr: Serum creatinine; BUN: Blood urea nitrogen; MN: Membranous nephropathy; MN-I: Stage I idiopathic membranous nephropathy; MN-II: Stage II idiopathic membranous nephropathy; m-MsPGN: Mild mesangioproliferative glomerulonephritis; MCD: Minimal change disease; PBS: Phosphate-buffered saline.

## Competing interest

The authors declare that they have no competing interests.

## Authors’ contribution

RZ and ZZ designed the study, performed research and wrote the first draft of the manuscript, JL and LQ participated in its research and the sequence alignment. FZ helped to draft the final version of the manuscript. All authors read and approved the final manuscript.
